# Plastic Gold

**Published:** 1857-04

**Authors:** 


*Plastic Gold*—The senior editor has recently received from M. Weber, dentist of Paris, a preparation of gold for filling teeth, designated by the above name. He has not, however, had an opportunity of testing its working qualities sufficiently to enable him to say much with regard to its value as compared with other preparations now in use. In appearance it does not differ much from sponge and crystalline gold. It is denser, and is less liable, he thinks, to crumble; or at least, a single experiment, which he made with it seemed to justify such an opinion. He intends to give it a fair trial as soon as he can find leisure to do so. He will also send some of it to two or three of his professional friends who are most favorable to the use of crystalline gold and have had most experience in its use.

In the communication which accompanied the "plastic gold," the writer says, "of late years one of the improvements which has most occupied the attention of the profession, is the stopping of decayed teeth. The preparers of gold for this purpose have followed in the same direction. Beaten gold has been considerably improved; then came sponge and crystalline gold. Spongy gold did not answer perfectly, and of crystalline gold I have only heard the name, and, therefore, can say nothing about it. Spongy gold is too friable and requires too great a volume to fill a carious tooth, even of moderate size; it also takes too much time, and after all, gives but indifferent results."

"I have long been endeavoring to discover a way of preparing gold which should be both spongy and ductile, and at the same time of sufficient consistence not to loose more than half or two-thirds of its value. Many of my friends confess that I have completely succeeded."

It is most sincerely to be hoped that M. Weber has succeeded in his wishes, and that it may prove to be better than any of the preparations heretofore used. The senior editor hopes to be able to give in the next number of the Journal, the result, not only of his own experiments, but also those of other members of the profession, trusting they may be as favorable as they seem to have been with the inventor, but whether they will be or not he is entitled to the thanks of his professional brethren for his efforts to produce a better preparation than those now in use. In the mean time, the senior editor begs to assure him that his wishes, as expressed in the communication which he was so kind as to address to him, shall be complied with.

